# Deoxynivalenol enhances IL-1ß expression in BV2 microglial cells through activation of the NF-?B pathway and the ASC/NLRP3 inflammasome

**DOI:** 10.17179/excli2018-1974

**Published:** 2019-06-11

**Authors:** Ilandarage Menu Neelaka Molagoda, Seunghun Lee, Rajapaksha Gedara Prasad Tharanga Jayasooriya, Cheng-Yung Jin, Yung Hyun Choi, Gi-Young Kim

**Affiliations:** 1 Department of Marine Life Sciences, Jeju National University, Jeju 63243, Republic of Korea; 2 Department of Bioprocess Technology, Faculty of Technology, Rajarata University of Sri Lanka, Mihintale 50300, Sri Lanka; 3 School of Pharmaceutical Sciences, Institute of Drug Discovery and Development, Key Laboratory of Advanced Pharmaceutical Technology, Ministry of Education of Chain, Zhengzhou University, Zhengzhou 450001, PR China; 4 Department of Biochemistry, College of Oriental Medicine, Dong-Eui University, Busan 47227, Republic of Korea

**Keywords:** deoxynivalenol, IL-1beta, nuclear factor-kappaB, inflammasome

## Abstract

Deoxynivalenol (DON) is one of the most common fungal toxins that contaminate food grains and cereal-derived products. However, it is unknown whether DON stimulates IL-1β expression through the activation of the nuclear factor-κB (NF-κB) pathway and the ACS/NLRP3 inflammasome. In this study, we found that high concentrations of DON (above 800 nM) decreased relative cell viability; however, no significant population of apoptotic sub-G_1_ cells was observed. DON also upregulated *IL-1β* expression from between 0.5 h and 6 h after treatment, and enhanced the nuclear localization of the NF-κB subunits, p50 and p65. NF-κB inhibitors, pyrrolidinedithiocarbamate and PS1145, significantly suppressed the DON-induced *IL-1β* expression, which indicated that DON increased *IL-1β* expression through the activation of NF-κB. In addition, marked secretion of IL-1β protein occurred in the presence of DON at 24 h, and a caspase-1 inhibitor suppressed DON-mediated IL-1β secretion, which suggested that caspase-1 induced the cleavage of pro-IL-1β to lead the secretion of its active form. Thus, components of the inflammasome, such as ASC and NLRP3, significantly increased by DON treatment; in addition, the knockdown of *ASC* and *NLRP3* markedly downregulated DON-induced IL-1β secretion, but not *IL-1β* gene expression, which indicated that DON promoted IL-1β secretion through the ASC/NLRP3 inflammasome. Collectively, the data suggested that DON induced IL-1β expression in BV2 microglial cells through the activation of the NF-κB signaling pathway and the subsequent upregulation of the ASC/NLRP3 inflammasome. Therefore, DON may induce inflammatory diseases or disorders by activating IL-1β expression.

## Introduction

Inflammation is regarded as an ordinary and essential component of the response to an infection and injury (Coussens and Werb, 2002[9]). It includes a sequence of complex, interrelated events that lead to the recruitment of phagocytes, the elimination of harmful particles and, finally, the initiation of tissue repair (Budai et al., 2013[5]; Zhong et al., 2016[44]). A network of cellular components, including inflammatory cytokines, governs this particular sequence of events. Interleukin-1β (IL-1β) is known as the major inflammatory mediator among these inflammatory cytokines (Tang et al., 2012[37]; Zhong et al., 2016[44]). The secretion of IL-1β leads to the activation of various cell types, including phagocytes, and epithelial and endothelial cells, and assists the activation and polarization of T lymphocytes, and enhances the expression of further pro-inflammatory cytokines, such as IL-6 and TNF-α (Dinarello, 2009[11]). IL-1β is also considered as an important pro-inflammatory cytokine in the brain and plays a critical role in the progression of neuroinflammation, which is a well-known factor in the pathogenesis of neurodegenerative diseases such as Alzheimer's disease, Parkinson's disease, and multiple sclerosis (Lee et al., 2016[23]).

Microglial cells are the resident macrophages in the brain and their secretion of IL-1β is strongly dependent on the multiprotein complex known as the inflammasome (Gustin et al., 2015[17]). Inflammasome plays an important role in the regulation of innate immunity and the inflammatory response (Ni et al., 2015[25]). To date, four distinct inflammasomes (NLRP1, NLRP3, NLRP4, and AIM) have been identified and they sense intracellular danger signals via NOD-like receptors that recognize pathogen-associated molecular patterns (Tang et al., 2012[37]). One of the most intensively studied inflammasomes is the NOD-like receptor family pyrin domain-containing 3 (NLRP3) inflammasome (Ni et al., 2015[25]), which contains an NLRP3 sensor, an apoptosis-associated speck-like protein containing a caspase recruitment domain (ASC) adaptor, and the caspase-1 enzyme (Schroder et al., 2010[33]).

Substantial evidence indicates that the NLRP3 inflammasome is primed by two main signals, termed primary and secondary signals. The primary signals trigger the synthesis of pro-IL-1β and NLRP3 through the transcriptional induction of NF-κB (Jin and Flavell, 2010[20]; Kufer et al., 2006[21]; Qu et al., 2017[29]; Zhang et al., 2016[43]); meanwhile, the secondary signals lead to oligomerization, caspase-1-dependent cleavage, and the subsequent release of biologically active IL-1β (Bauernfeind et al., 2009[3]; Ni et al., 2015[25]; Phan et al., 2015[28]). The secondary signals are composed of a group of chemically and biologically unrelated molecules, including pathogen-associated molecular patterns or damage-associated molecular patterns (Petrilli et al., 2007[27]; Schroder et al., 2010[33]). For example, ATP induces NLRP3 activation through the stimulation of the purinergic receptor, P2X ligand-gated ion channel 7 (P2X7), which induces K^+^ efflux (Jin and Flavell, 2010[20]). Millimolar concentrations of ATP are required for P2X7-mediated caspase-1 activation by activating NRLP3 inflammasome. High concentrations of ATP are not normally found in the *in vivo* extracellular environment, although they may be achieved under the circumstances of cell lysis, injury, or inflammation.

DON is a member of the family of trichothecene mycotoxins that is primarily found in cereal grains such as wheat, barley, and maize (Yin et al., 2016[42]). It is a secondary metabolite of several fungi, including *Fusarium*,* Mycothecium*,* Trichorderma*,* Trichothecium*,* Stachybotrys*,* Verticinosporium*, and *Chephalosporium *species (Akbari et al., 2016[1]; Wu et al., 2017[41]). DON affects the gastrointestinal, reproductive, and neuroendocrine systems, particularly the immune system; these changes induce emesis, diarrhea, and hemorrhage, and reduce the reproductive capacity of humans and animals (Deng et al., 2016[10]). Choi and his colleagues (2009[6]) demonstrated that DON rapidly activated mitogen-activated protein kinases under *in* *vitro* and *in* *vivo* conditions, which drive the upregulated mRNA and protein expression of inflammation-related genes, such as cytokines, chemokines, and cyclooxygenase-2 (He et al., 2013[18]). Girardet et al. (2011[15]) reported that DON could increase pro-inflammatory cytokines in the central nervous system concomitant with sickness-like behavior , which means that DON induces disturbance of the central nervous system by unbalancing pro-inflammatory cytokine production. Nevertheless, it is not known whether DON induces the activation of the inflammasome in microglia. Therefore, in this study, we investigated whether DON was involved in IL-1β expression and secretion in BV2 microglial cells through the activation of NF-κB and the inflammasome.

## Materials and Methods

### Reagents and antibodies

LPS, 3-(4,5-dimethylthiazol-2-yl)-2,5-diphenyl-tetrazolium bromide (MTT), pyrrolidinedithiocarbamate (PDTC), and PS1145 were purchased from Sigma-Aldrich (St. Louis, MO). The antibodies against IL-1β, caspase-1, β-actin, ASC, and NLRP3 were obtained from Santa Cruz Biotechnology (Santa Cruz, CA). Dulbecco's modified Eagle's medium (DMEM), fetal bovine serum (FBS), and antibiotic mixtures were obtained from WelGENE Inc. (Daegu, Republic of Korea). Other chemicals were purchased as Sigma grades.

### Cell culture and viability

Murine BV2 microglial cells (from E.H. Joe, Ajou University School of Medicine, Suwon, Republic of Korea) were cultured in DMEM supplemented with 10 % FBS in a CO_2_ incubator with a humidified atmosphere containing 5 % CO_2_ at 37 °C. Cell viability was determined by colorimetric MTT assay. Briefly, BV2 microglial cells (1 × 10^5^ cells/ mL) were treated with various concentrations (0-2000 nM) of DON. After 24 h incubation, the cells were incubated with MTT solution (0.5 mg/mL) for 30 min at 37 °C. Insoluble formazan was dissolved in DMSO and observed by monitoring the signal at 540 nm using a microplate reader (Thermo Electron Corp., Marietta, OH).

### Reverse transcriptase polymerase chain reactions (RT-PCR)

Total RNA was extracted using Easy-blue reagent (iNtRON Biotechnology, Sungnam, Republic of Korea) according to the manufacturer's instructions. Genes of interest were amplified from cDNA that was reverse-transcribed from 1 μg total RNA using the One-Step RT-PCR Premix (iNtRON Biotechnology). The specific primers for *caspase-1* (forward 5'-CTG ACT GGG ACC CTC AAG-3' and reverse 5'-CCT CTT CAG AGT CTC TTA CTG-3'), *IL-1β* (forward 5'- GCC CAT CCT CTG TGA CTC AT-3' and reverse 5'- AGG CCA CAG GTA TTT TGT CG-3'), *NLRP3* (forward 5'- TCG CAG CAA AGA TCC ACA CAG-3' and reverse 5'- ATT ACC CGC CCG AGA AAG G-3'), *ASC *(forward 5'- GCA ACT GCG AGA AGG CTA T-3' and reverse 5'- CTG GTC CAC AAA GTG TCC TG-3'), *GAPDH* (forward 5'- AGG TCG GTG TGA ACG GAT TTG-3' and reverse 5'-TGT AGA CCA TGT AGT TGA GGT CA-3'). The following PCR conditions were applied: for *NLRP3*: 30 cycles of denaturation at 94 °C for 30 s, annealing at 61 °C for 30 s and extended at 72 °C for 30 s; for *IL-1β*: 30 cycles of denaturation at 94 °C for 30 s, annealing at 64 °C for 30 s and extended at 72 °C for 30 s; for* caspase-1*: 30 cycles of denaturation at 94 °C for 30 s, annealing at 56 °C for 30 s and extended at 72 °C for 30 s; for *ASC*: 59 cycles of denaturation at 94 °C for 30 s, annealing at 64 °C for 30 s and extended at 72 °C for 30 s; for *GAPDH*: 27 cycles of denaturation at 94 °C for 30 s, annealing at 58 °C for 30 s and extended at 72 °C for 30 s. *GAPDH* was used as an internal controller to evaluate the relative expression of *IL-1β, caspase-1, NLRP3 *and* ASC*.

### Western blot analysis

BV2 microglial cells were seeded at the density of 1 × 10^5 ^cells/mL and treated with the indicated concentrations of DON or 1 mM ATP and 100 ng/mL LPS. After 24 h-incubation, total cell extracts were prepared using a PROPREP protein extraction solution (iNtRON Biotechnology). Briefly, the PROPREP solution was treated to the cells on the ice for 30 min and lysates were centrifuged at 14,000 × *g* for 10 min to obtain the supernatants. In a parallel experiment, cytoplasmic and nuclear extracts were prepared from the cells using NE-PER nuclear and cytosolic extraction reagents (Pierce, Rockford, IL). Protein concentrations were determined using a Bio-Rad protein assay kit (Bio-Rad, Hercules, CA). The samples were stored at -80 °C or immediately used for Western blot analysis after the extraction. The proteins were separated on SDS-polyacrylamide gels and transferred to nitrocellulose membranes (Schleicher & Schuell, Keene, NH). The transferred proteins were detected using an enhanced chemiluminescence detection system (Amersham, Arlington Heights, IL).

### Flow cytometric analysis

Apoptotic sub-G_1_ phase was analyzed by Muse^TM^ Cell Cycle Assay Kit (EMD Millipore Corp., Hayward, CA). Briefly, BV2 microglial cells (1 × 10^5 ^cells/mL) were treated with various concentrations of DON for 24 h. The cells were washed with phosphate-buffered saline (PBS) and stained according to the manufacturer´s protocol. The levels of apoptotic cells with sub-G_1_ DNA were determined as a percentage of the total number of cells using Muse^TM^ cell cycle analyzer (EMD Millipore Corp.). The results were analysed using Flowing Software (http://flowingsoftware.btk.fi/).

### ELISA

The cell free supernatants were collected from cultures 24 h after DON treatment and assayed for concentration of human IL-1β using Ready-set-go ELISA kit (eBioscience, San Diego, CA). The test was performed according to the ready-set-go protocol.

### Transfection of siRNA

BV2 microglial cells were seeded on a 12-well plate at a density of 1 × 10^5 ^cells/mL and transfected *ASC* and *NLRP3*-specific silencing RNA (siRNA, Santa Cruz Biotechnology) for 48 h. For each transfection, 450 μL growth medium was added to 20 nM siRNA duplex with the transfection reagent G-Fectin (Genolution Pharmaceuticals Inc., Seoul, Republic of Korea) and the entire mixture was added gently to the cells.

### Statistical analysis

All data were derived from at least three independent experiments. The images were visualized with Chemi-Smart 2000 (Vilber Lourmat, Cedex, France). Images were captured using Chemi-Capt (Vilber Lourmat) and transported into Adobe Photoshop (version 8.0). All bands were quantified by Image J software (https://imagej.net). All data of RT-PCR and Western blots were statistically analyzed by Sigma plot 12.0 software. All data are presented as mean ± standard error (SE). Significant differences between the groups were determined with one-way analysis of variance (ANOVA) with Bonferroni′s test. Values of ***, *p* < 0.001, **, *p* < 0.01, and *, *p* < 0.05 were accepted as an indication of statistical significance.

## Results

### Low concentrations of DON are not cytotoxic to BV2 microglia cells

**Figure 1:**
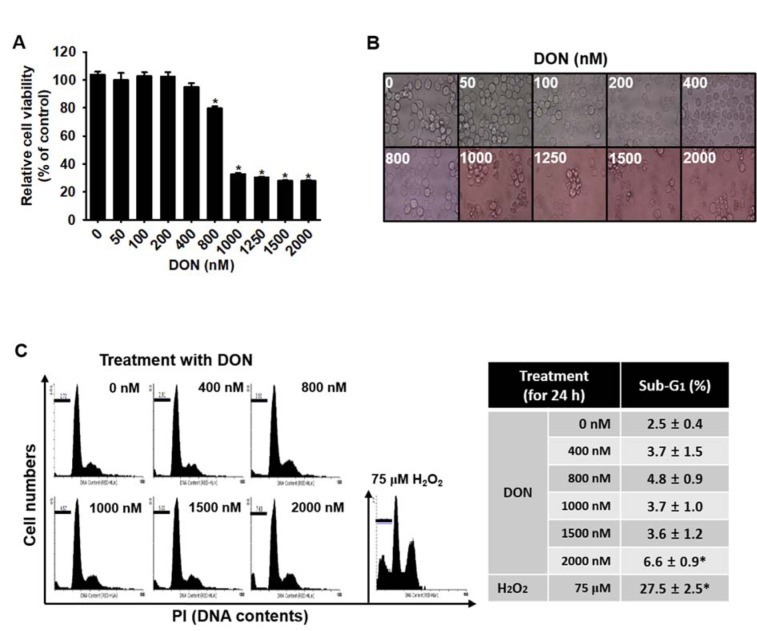
Deoxynivalenol (DON) exhibits a slight influence on the viability of BV2 microglial cells. BV2 microglial cells were seeded at a density of 1 × 10^5^ cells/mL and incubated with various concentrations of DON for 24 h. (A) The relative cell viability was determined by using an MTT assay. (B) The morphology of cells was examined under a light microscope (× 400 magnification) and analyzed by using ToupView software. (C) The percentages of sub-G1 DNA content were analyzed by flow cytometry and the images are representative analyses. Data from three independent experiments are expressed as the overall mean ± S.E. Statistical significance was determined by one-way ANOVA *, p < 0.05 vs. untreated control.

To assess the effect of DON on the viability of BV2 microglial cells, we treated the cells with the indicated concentrations of DON (up to 2000 nM) for 24 h and then performed an MTT assay. A significant reduction in cell viability was observed after treatment with 1000 nM DON and no significant changes were observed below 400 nM DON (Figure 1A(Fig. 1)). At 800 nM DON, a moderate decrease in cell viability occurred, to approximately 80 % of the control. Next, the direct cytotoxic effects of DON were measured through an analysis of the morphological changes in the cells and the sub-G_1_ population. We did not observe notable morphological changes following treatment with up to 800 nM DON; however, higher concentrations (over 1000 nM) of DON induced a slight shrinkage of cells (Figure 1B(Fig. 1)). In addition, flow cytometric data showed that no more cells in the apoptotic sub-G_1_ phase were observed in the presence of up to 1500 nM DON compared with that in the H_2_O_2_-treated group; however, 2000 nM DON was observed to induce a slight increase of the sub-G_1_ population, of up to 6.6 % ± 0.9 % (Figure 1C(Fig. 1)). Collectively, these data indicated that low concentrations (≤ 800 nM) of DON were not cytotoxic to BV2 microglial cells.

### DON induces the expression of the IL-1β gene in BV2 microglia cells through activation of the NF-κB signaling pathway

**Figure 2:**
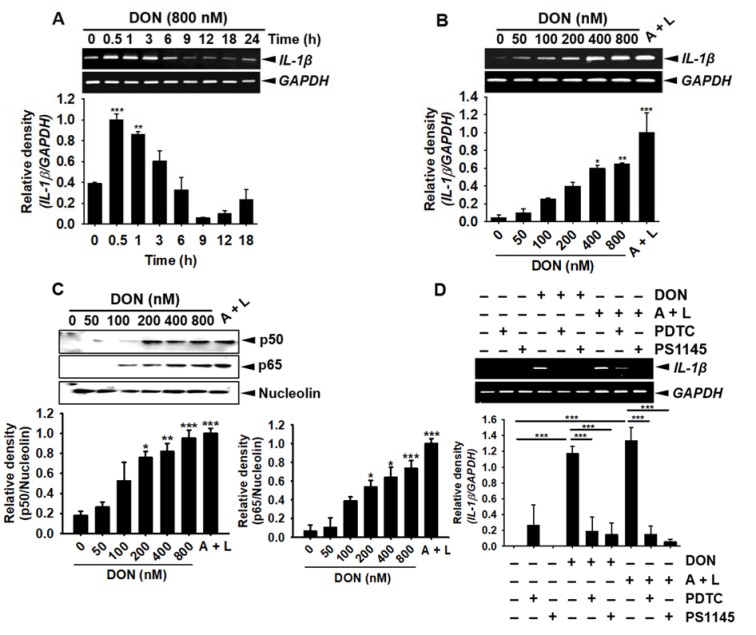
DON induces IL-1β expression in BV2 microglia cells through the activation of NF-κB activity. (A) BV2 microglial cells (1 × 10^5^ cells/mL) were treated with 800 nM DON and harvested at the indicated time points. Total cellular RNA was subjected to RT-PCR and the PCR products were separated on a 2 % agarose gel. (B) The cells were treated with the indicated concentrations of DON or 1 mM ATP and 100 ng/mL LPS for 1 h. The extracted mRNA was then subjected to RT-PCR and the PCR products were separated on a 2% agarose gel. (C) In a parallel experiment, the cells were treated with the indicated concentrations of DON or 1 mM ATP and 100 ng/mL LPS for 30 min, after which the nuclear compartment was purified and Western blotting for p50 and p65 was performed; nucleolin was used as a control nuclear protein. (D) For the functional analysis of NF-κB, BV2 microglial cells were pre-incubated with 10 µM PDTC and 10 µM PS1145 for 1 h and then treated with 800 nM DON or 1 mM ATP and 100 ng/mL LPS. Total cellular RNA was subjected to RT-PCR analysis for IL-1β expression. The data from three independent experiments were expressed as the overall mean ± S.E. Statistical significance was determined by one-way ANOVA ***, p < 0.001, **, p < 0.01 and *, p < 0.05 vs. untreated control). A + L; treatment with 1 mM ATP and 100 ng/mL LPS.

To find the changes in the gene expression of *IL-1β* over time, 800 nM DON-treated samples were collected at the indicated time points and analyzed by RT-PCR. *IL-1β *was highly expressed at 0.5-3 h after treatment with DON (Figure 2A(Fig. 2)). To assess the DON concentration dependency of *IL-1β* gene expression, we treated the cells with the indicated concentrations of DON for 1 h. *IL-1β *gene expression was markedly upregulated after the incubation of the cells with over 400 nM and 800 nM DON, similar to the effects of treatment with 1 mM ATP and 100 ng/mL LPS (ATP/LPS) (Figure 2B(Fig. 2)). Low concentrations of DON (below 400 nM) resulted in a moderate increase in *IL-1β* expression. We then found that the NF-κB heterodimer subunits, p50 and p65, were markedly upregulated in the nuclear compartments after 30 min of DON incubation (Figure 2C(Fig. 2)). Next, to determine the effect of NF-κB on DON-mediated *IL-1β* gene expression, we pretreated the cells with the NF-κB inhibitors, PDTC and PS1145, for 1 h before DON treatment was started. The pre-incubation of PDTC and PS1145 significantly inhibited the upregulation of *IL-1β *gene expression induced by DON or ATP/LPS, which confirmed that DON increased the NF-κB-mediated expression of *IL-1β* (Figure 2D(Fig. 2)). Collectively, these results indicated that DON induced the expression of *IL-1β* in BV2 microglia cells through the activation of the NF-κB signaling pathway.

### DON increases the secretion of active IL-1β protein from BV2 microglial cells

**Figure 3:**
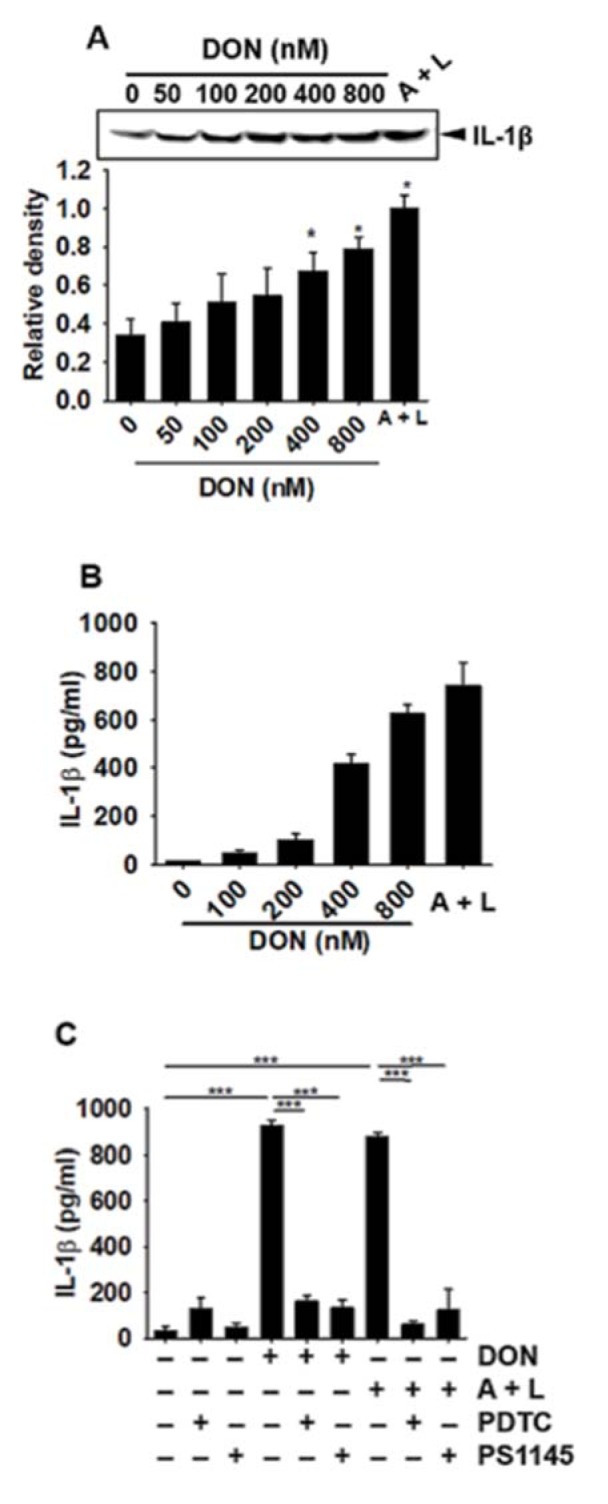
DON increases the secretion of active IL-1β in BV2 microglia cells. BV2 microglial cells (1 × 10^5^ cells/mL) were treated with the indicated concentrations of DON or 1 mM ATP and 100 ng/mL LPS for 24 h and the culture media was collected. Western blotting (A) and ELISA (B) were performed to measure active IL-1β secretion. (C) In a parallel experiment, BV2 microglial cells were pre-treated with the NF-κB inhibitors, PDTC (10 µM) and PS1145 (10 µM) for 1 h, followed by treatment with 800 nM DON or 1 mM ATP and 100 ng/mL LPS. Active IL-1β secretion was quantified by ELISA. Data from three independent experiments are expressed as the overall mean ± S.E. Statistical significance was determined by one-way ANOVA ***p < 0.001 and *, p < 0.05). A + L; treatment with 1 mM ATP and 100 ng/mL LPS.

As DON was shown to upregulate *IL-1β* expression, we then focused on the secretion of active IL-1β that was induced by the cleavage of pro-IL-1β. Active IL-1β in culture media was assessed by Western blotting and ELISA at 24 h after treatment with DON or ATP and LPS. As shown in Figure 3A(Fig. 3) and Figure 3B(Fig. 3), DON concentration-dependently upregulated IL-1β secretion. Above 400 nM DON significantly upregulated extracellular IL-1β. However, the NF-κB inhibitors, PDTC and PS1145, decreased the DON-induced secretion of IL-1β (Figure 3C(Fig. 3)). All results suggested that DON upregulated IL-1β secretion in BV2 microglia cells as a resulting of NF-κB activation.

### DON enhances IL-1β secretion through activation of caspase-1

**Figure 4:**
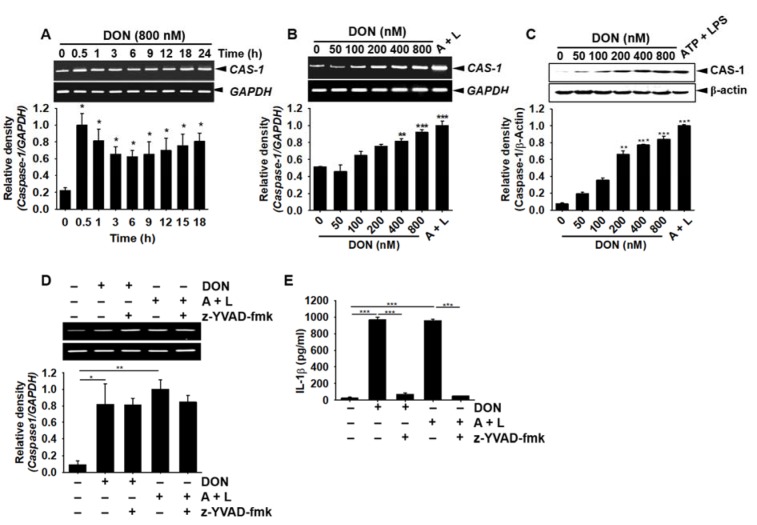
DON increases the expression of caspase-1 in BV2 microglial cells, leading to the secretion of active IL-1β. (A) BV2 microglial cells were seeded at a density of 1 × 10^5^ cells/mL, treated with 800 nM DON, and harvested at the indicated time points. RT-PCR analysis was conducted to assess the time course for *caspase-1* expression. (B) The cells were treated with the indicated concentrations of DON or 1 mM ATP and 100 ng/mL LPS. The effect of DON on *caspase-1* expression in BV2 microglial cells was assessed by RT-PCR. (C) In a parallel experiment, Western blotting for caspase-1 was performed. (D) Cells were pretreated with z-YVAD-fmk (10 µM) 2 h before treatment with DON or 1 mM ATP and 100 ng/mL LPS, and *caspase-1* expression was determined by RT-PCR. (E) Caspase-1-dependent IL-1β secretion was elucidated ELISA of the cell culture supernatant after incubation for 24 h with a caspase-1 inhibitor, z-YVAD-fmk. Data from three independent experiments are expressed as the overall mean ± S.E. Statistical significance was determined by one-way ANOVA ***, *p* < 0.001, **, *p* < 0.01, and *, *p *< 0.05 vs. untreated control). CAS-1; caspase-1. A + L; treatment with 1 mM ATP and 100 ng/mL LPS.

The cleavage of pro-IL-1β into its active form requires the manipulation of active caspase-1. Therefore, we evaluated the effect of DON on caspase-1 expression in BV2 microglial cells. DON (800 nM) triggered the expression of *caspase-1* from 0.5 h and sustained this expression to 24 h (Figure 4A(Fig. 4)). DON (≥ 100 nM) also increased the expression of *caspase-1* in a concentration-dependent manner (Figure 4B(Fig. 4)). Intracellular active caspase-1 protein was significantly upregulated by DON incubation in a concentration-dependent manner (Figure 4C(Fig. 4)). When the cells were pre-treated with a caspase-1 inhibitor, z-YVAD-fmk, the expression of *IL-1β* was still observed in the presence of DON or ATP and LPS (Figure 4D(Fig. 4)); however, the expression of extracellular active IL-1β was significantly downregulated (Figure 4E(Fig. 4)), which indicated that DON-induced caspase-1-cleaved pro-IL-1β to induce the secretion of active IL-1β. These results suggested that DON increased active caspase-1 in BV2 microglial cells, which subsequently cleaved pro-IL-1β into active IL-1β.

### DON upregulates the ASC/NLRP3 inflammasome, which stimulates IL-1β secretion through the activation of caspase-1

**Figure 5:**
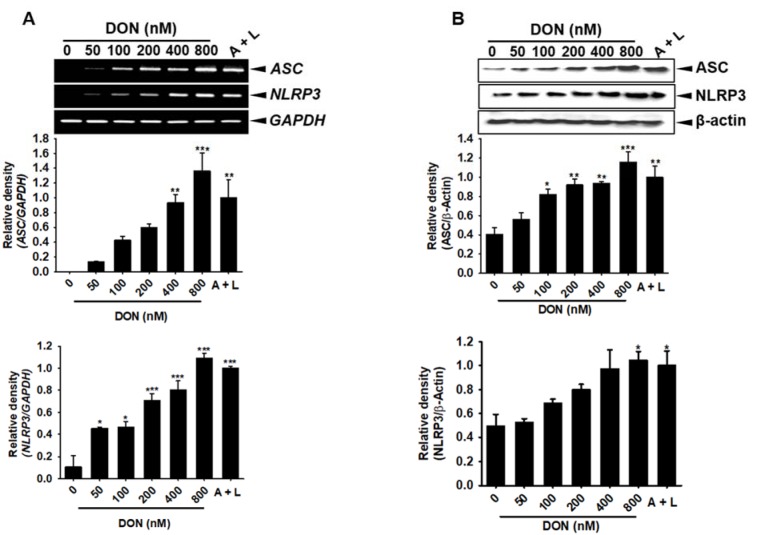
DON upregulates ASC and NLRP3 expression. BV2 microglial cells were seeded at a density of 1 × 10^5^ cells/mL and treated with the indicated concentrations of DON or 1 mM ATP and 100 ng/mL LPS. (A) At 1 h after the administration of DON, the effect on *ASC* and *NLRP3* expression was assessed by RT-PCR. (B) The cytosolic fraction of BV2 microglial cell lysate was used to assess ASC and NLRP3 protein expression after 24 h. The data from three independent experiments are expressed as the overall mean ± S.E. Statistical significance was determined by one-way ANOVA ***, *p* < 0.001, **, *p* < 0.01, and *, *p *< 0.05 vs. untreated control). A + L; treatment with 1 mM ATP and 100 ng/mL LPS.

**Figure 6:**
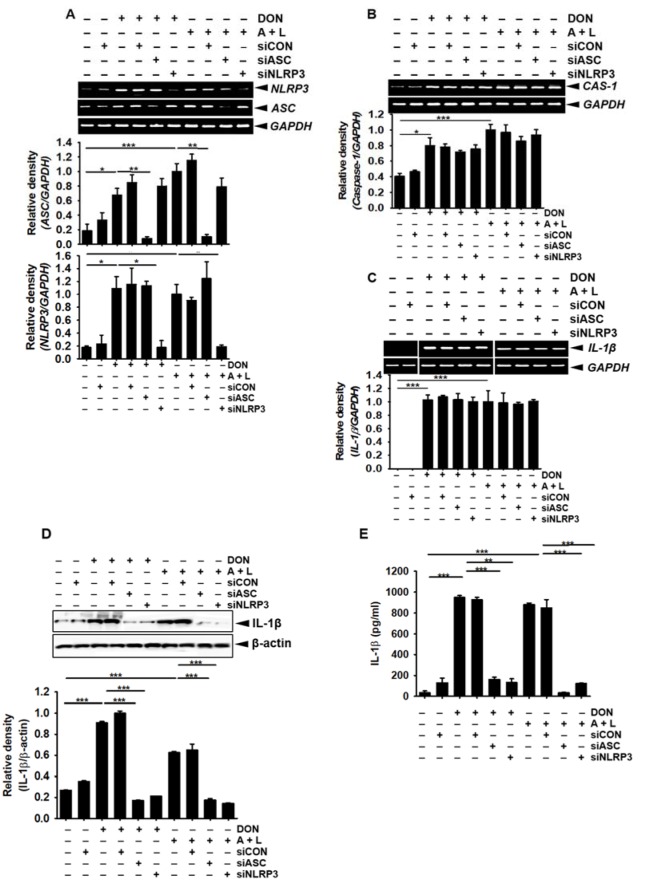
DON enhances capase-1-mediated active IL-1β through the activation of the ASC/NLRP3 inflammasome. BV2 microglial cells were seeded at the density of 1 × 10^5^ cells/mL overnight and then transfected with *siASC* and *siNLRP3* for 48 h. The cells were treated with DON (800 nM), or 1 mM ATP and 100 ng/mL LPS. (A) The effect of DON on *ASC* and *NLRP3* expression was assessed by RT-PCR 1 h after treatment with DON, or ATP and LPS. (B and C) In a parallel experiment, *caspase-1* (B) and *pro-IL-1β* (C) expression was detected by RT-PCR at samples taken 1 h after treatment. (D and E) The protein expression of active caspase-1 (D) and IL-1β (E) expression was detected by using Western blotting and ELISA at 24 h, respectively. The data from three independent experiments are expressed as the overall mean ± S.E. Statistical significance was determined by one-way ANOVA ***, *p* < 0.001, **, *p* < 0.01, and *, *p *< 0.05 vs. untreated control). CAS-1; caspase-1. A + L; treatment with 1 mM ATP and 100 ng/mL LPS.

The cleavage of pro-caspase-1 into active caspase-1 requires the formation of an inflammasome complex. Therefore, we assessed the effect of DON on inflammasome complex formation in BV2 microglial cells. As expected, DON concentration-dependently increased the gene (Figure 5A(Fig. 5)) and protein (Figure 5B(Fig. 5)) expression of ASC and NLRP3. In addition, the transient knockdown of *ASC* and *NLRP3* significantly suppressed the expression of *ASC* and *NLRP3* induced by DON (Figure 6A(Fig. 6)); however, the expression of DON-induced *caspase-1* (Figure 6B(Fig. 6)) and *IL-1β* (Figure 6C(Fig. 6)) was sustained, which suggested that the gene expression of *caspase-1* and *IL-1β* was not regulated by the ASC/NLRP3 inflammasome. Thus, active caspase-1 and IL-1β were markedly increased by treatment with DON; however, this strong expression was diminished in the cells transfected with *ASC* and *NLRP3* siRNA (Figure 6D(Fig. 6) and Figure 6E(Fig. 6)). Collectively, these results suggested that DON upregulated the expression of the ASC/NLRP3 inflammasome complex, which subsequently induced active caspase-1 and led to the secretion of active IL-1β.

For more results see Supplementary data.

## Discussion

DON is one of the most common food-associated mycotoxins, particularly in cereals and cereal-derived products, and possesses cell survival, activation, and inflammation at low concentrations (nM) (Maresca, 2013[24]). As DON also contributes to brain tumor progression (Varini et al., 2012[39]), pain hypersensitivity (Ren and Dubner, 2008[31]), and alterations in learning and memory consolidation (Gibbs et al., 2008[14]), we focused on the pro-inflammatory effect of DON in BV2 microglial cells through the activation of the inflammasome. Microglial cells are responsible for the maintenance of brain homeostasis and the survival of neurons (Salmina, 2009[32]). However, the chronic hyperactivation of microglial cells results in neuroinflammation, whereas hypoactivation is associated with an increased sensitivity of the brain to infections (Razafimanjato et al., 2011[30]). Many previous data showed that DON (100 - 250 ng/ml) is effective to pro-inflammatory cytokines *in* *vitro* (Bonnet et al., 2012[4]; Pestka and Amuzie, 2008[26]; Sugita-Konishi and Pestka, 2001[35]). Our data also showed that 400 nM and 800 nM DON (approximately 120 ng/ml and 240 ng/ml, respectively) significantly increased IL-1β expression by activating the NF-κB pathway and NLRP3 inflammasome. Above 1000 nM DON (approximately 300 ng/ml) also increased IL-1β expression, but significantly decreased cell growth without any cytotoxicity. In addition, DON administration (10-20 mg/Kg) was severely increased central inflammation and sickness-like behavior in mouse model (Girardet et al., 2011[15]), which means that DON also give rise to brain disorders; however, we still don't know how much DON can penetrate blood-vessel barrier.

IL-1β, a potent pro-inflammatory cytokine released by many different immune cells such as microglia and macrophages, is essential for the host-response during infection and inflammation (Fogal and Hewett, 2008[13]); however, an accumulation of evidence has demonstrated that excessive IL-1β secretion is a major contributor to neuroinflammation and Alzheimer's disease (Shaftel et al., 2008[34]). There are two signaling steps associated with active IL-1β secretion. The first signal is associated with NF-κB priming for the expression of *IL-1β* (Cogswell et al., 1994[7]) and the second signal is controlled by the inflammasome complex formation for the maturation of pro-IL-1β into active IL-1β (Barker et al., 2011[2]). *IL-1β* expression induced by NF-κB is required for the assistance of caspase-1, which transforms pro-IL-1β to mature or active IL-1β, resulting in its secretion from cells (Lamkanfi, 2011[22]). The combination of pro-caspase-1, ASC, and NLRP3 is known as the NLRP3 inflammasome complex, which ultimately leads to the activation of pro-caspase-1 for active caspase-1 and promotes the cleavage of pro-IL-1β to the secretion of mature or active IL-1β (Lamkanfi, 2011[22]). LPS is a TLR4 ligand that activates the NF-κB pathway and thereby upregulates *IL-1β* transcription (Grahames et al., 1999[16]). Extracellular ATP mediates a wide range of effects through action on the P2 receptors, which are classified as either P2X (ligand-gated ion channel) or P2Y (G protein-coupled receptor) receptors (Dubyak, 1991[12]). The binding of extracellular ATP into ionotropic P2X7 receptor leads the cell to form the NLRP3 inflammasome complex (Couillin et al., 2013[8]). In the current study, we found that DON induced the *IL-1β* expression and extracellular IL-1β secretion from BV2 microglia cells. Previous studies revealed that DON increased NF-κB activity in monocytes and T cells (Van De Walle et al., 2008[38]; Wong et al., 2002[40]), which induced inflammation; however, the possibility that DON could inhibit NF-κB activity was also found in activated macrophages treated with TNF-α or TLR ligands (Hirano and Kataoka, 2013[19]; Sugiyama et al., 2016[36]). We found that treatment of BV2 microglial cells with DON significantly induced the expression of ASC and NLRP3. However, the transient knockdown of *ASC* and *NLRP3* did not influence *caspase-1* or *IL-1β* expression, which indicated that ASC and NLRP3 did not regulate *caspase-1* or *IL-1β* expression. Nevertheless, the transient knockdown of *ASC* and *NLRP3* significantly decreased the protein synthesis of active caspase-1 and active IL-1β, which confirmed the critical roles of the active form of caspase-1 and IL-1β. These data indicated that DON induced neuroinflammation through enhanced IL-1β secretion mediated by the activation of NF-κB and the ASC/NLRP3 inflammasome.

Collectively, our results have demonstrated that DON upregulated *IL-1β* gene expression via the NF-κB pathway and promoted the ASC/NLRP3 inflammasome, which led to the cleavage of pro-IL-1β into active IL-1β and its secretion out of the cells. Although the brain permeability of DON may be different in humans and mice, DON exposure may also be a particular risk factor for neurological disorder, including brain diseases, in humans.

## Acknowledgement

The research was supported by the Program of National Research Foundation of Korea through the Ministry of Education.

## Conflict of interest

The authors declare that they have no conflict of interest.

## Supplementary Material

Supplementary data
