# Supplementary material for: Deoxynivalenol enhances IL-1ß expression in BV2 microglial cells through activation of the NF-?B pathway and the ASC/NLRP3 inflammasome
Source: EXCLI J. 2019 Jun 11;18:356–69. doi: 10.17179/excli2018-1974 (PMC6635726; doi:10.17179/excli2018-1974)
Supplement: Supplementary data [file EXCLI-18-356-s-001.pdf]

*EXCLI Journal 2019;18:356-369 – ISSN 1611-2156*
*Received: December 07, 2018, accepted: March 07, 2019, published: June 11, 2019*

## Supplementary data to:

# DEOXYNIVALENOL ENHANCES IL-1ß EXPRESSION IN BV2 MICROGLIAL CELLS THROUGH ACTIVATION OF THE NF-KB PATHWAY AND THE ASC/NLRP3 INFLAMMASOME

Ilandarage Menu Neelaka Molagoda[1], Seunghun Lee[1], Rajapaksha Gedara Prasad Tharanga Jayasooriya[2], Cheng-Yung Jin[3], Yung Hyun Choi[4], Gi-Young Kim[1,*]

1 Department of Marine Life Sciences, Jeju National University, Jeju 63243, Republic of Korea
2 Department of Bioprocess Technology, Faculty of Technology, Rajarata University of Sri Lanka, Mihintale 50300, Sri Lanka
3 School of Pharmaceutical Sciences, Institute of Drug Discovery and Development, Key Laboratory of Advanced Pharmaceutical Technology, Ministry of Education of Chain, Zhengzhou University, Zhengzhou 450001, PR China
4 Department of Biochemistry, College of Oriental Medicine, Dong-Eui University, Busan 47227, Republic of Korea

* Corresponding author: Prof. Gi-Young Kim, Department of Marine Life Sciences, Jeju National University, Jeju 63243, Republic of Korea, Tel: 82-64-754-3420, Fax: 82-64-754-3427, E-mail: immunkim@jejunu.ac.kr

http://dx.doi.org/10.17179/excli2018-1974

This is an Open Access article distributed under the terms of the Creative Commons Attribution License (http://creativecommons.org/licenses/by/4.0/).

EXCLI Journal 2019;18:356-369 – ISSN 1611-2156
Received: December 07, 2018, accepted: March 07, 2019, published: June 11, 2019

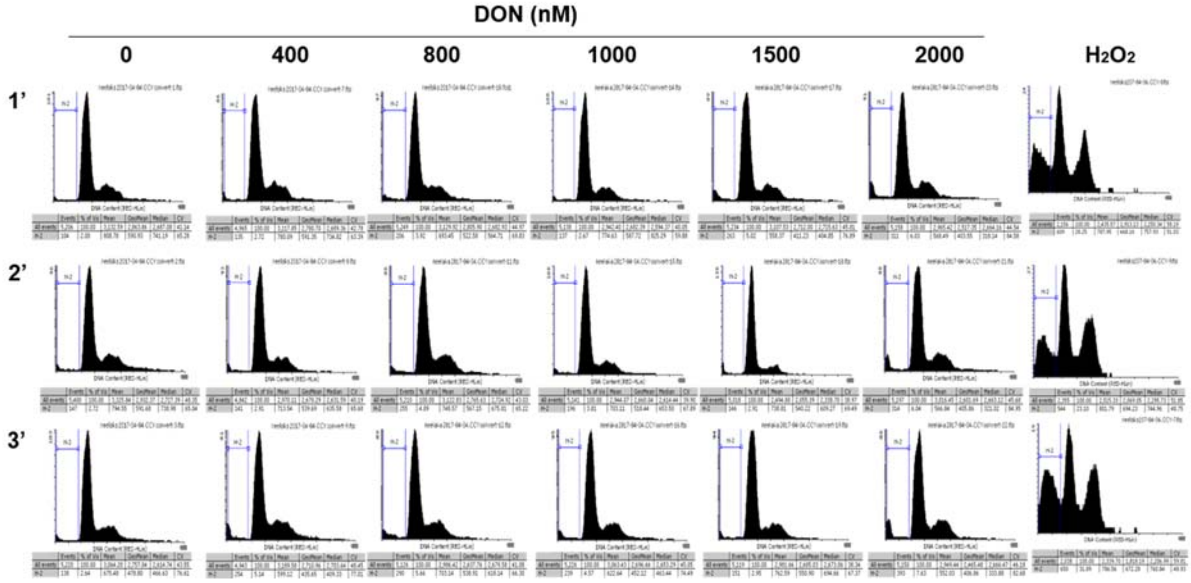


**Supplementary Figure 1:** These are supplementary data for Figure 1C. BV2 microglial cells were seeded at a density of 1 × $10^5$ cells/mL and incubated with various concentrations of DON for 24 h. The percentages of sub-$G_1$ DNA content were analyzed by flow cytometry and the images are representative analyses.

EXCLI Journal 2019;18:356-369 – ISSN 1611-2156
Received: December 07, 2018, accepted: March 07, 2019, published: June 11, 2019

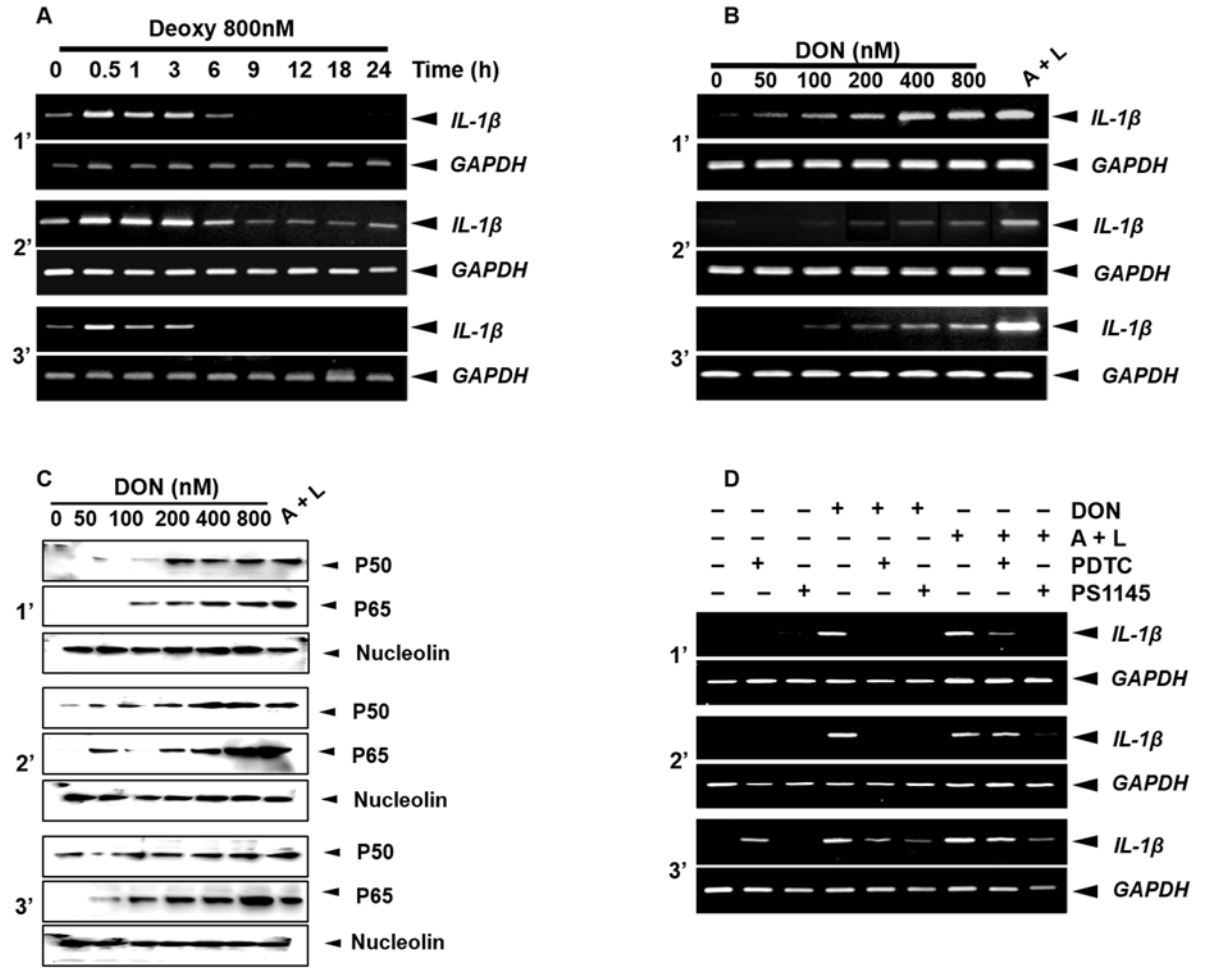


**Supplementary Figure 2:** These are all triplicate experiments for Figure 2. (**A**) BV2 microglial cells (1 × $10^5$ cells/mL) were treated with 800 nM DON and harvested at the indicated time points. Total cellular RNA was subjected to RT-PCR and the PCR products were separated on a 2 % agarose gel. (**B**) The cells were treated with the indicated concentrations of DON or 1 mM ATP and 100 ng/mL LPS for 1 h. The extracted mRNA was then subjected to RT-PCR and the PCR products were separated on a 2 % agarose gel. (**C**) In a parallel experiment, the cells were treated with the indicated concentrations of DON or 1 mM ATP and 100 ng/mL LPS for 30 min, after which the nuclear compartment was purified and western blotting for p50 and p65 was performed; nucleolin was used as a control nuclear protein. (**D**) For the functional analysis of NF-κB, BV2 microglial cells were pre-incubated with 10 µM PDTC and 10 µM PS1145 for 1 h and then treated with 800 nM DON or 1 mM ATP and 100 ng/mL LPS. Total cellular RNA was subjected to RT-PCR analysis for *IL-1β* expression.

EXCLI Journal 2019;18:356-369 – ISSN 1611-2156
Received: December 07, 2018, accepted: March 07, 2019, published: June 11, 2019

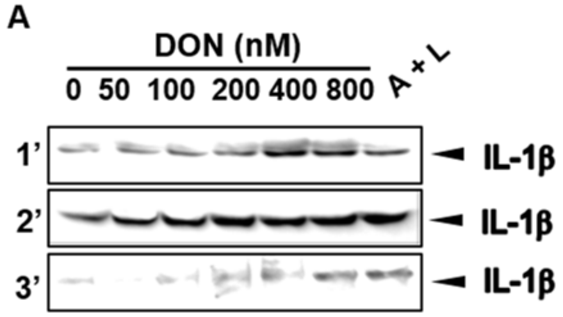


**Supplementary Figure 3:** These are all triplicate experiments for Figure 3A. BV2 microglial cells (1 × $10^5$ cells/mL) were treated with the indicated concentrations of DON or 1 mM ATP and 100 ng/mL LPS for 24 h and the culture media was collected. Western blotting analysis was performed to measure active IL-1β secretion.

EXCLI Journal 2019;18:356-369 – ISSN 1611-2156
Received: December 07, 2018, accepted: March 07, 2019, published: June 11, 2019

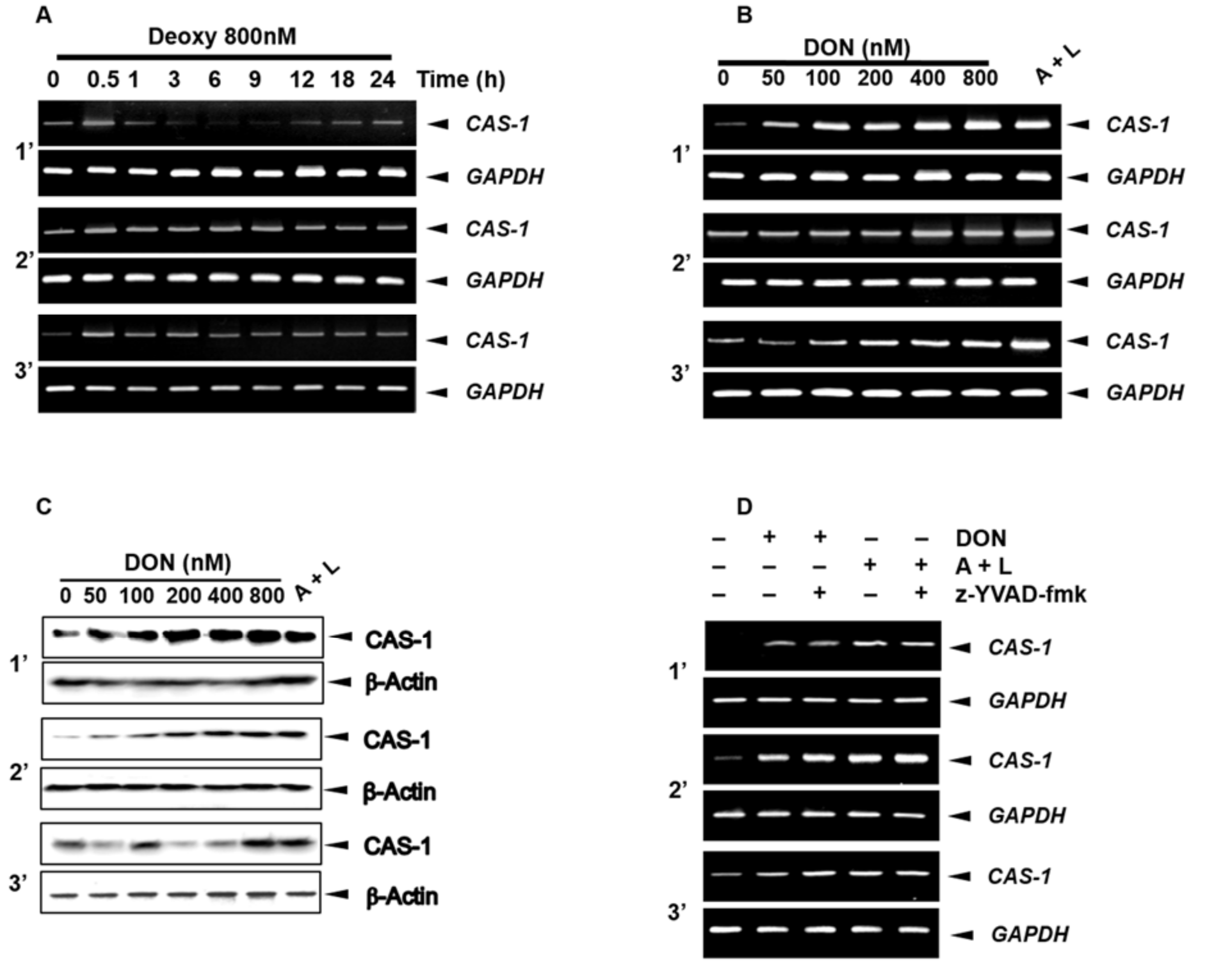


**Supplementary Figure 4:** These are all triplicate experiments for Figure 4. (**A**) BV2 microglial cells were seeded at a density of 1 × $10^5$ cells/mL, treated with 800 nM DON, and harvested at the indicated time points. RT-PCR analysis was conducted to assess the time course for *caspase-1* expression. (**B**) The cells were treated with the indicated concentrations of DON or 1 mM ATP and 100 ng/mL LPS. The effect of DON on *caspase-1* expression in BV2 microglial cells was assessed by RT-PCR. (**C**) In a parallel experiment, western blotting for caspase-1 was performed. (**D**) Cells were pretreated with z-YVAD-fmk (10 μM) 2 h before treatment with DON or 1 mM ATP and 100 ng/mL LPS, and *caspase-1* expression was determined by RT-PCR.

EXCLI Journal 2019;18:356-369 – ISSN 1611-2156
Received: December 07, 2018, accepted: March 07, 2019, published: June 11, 2019

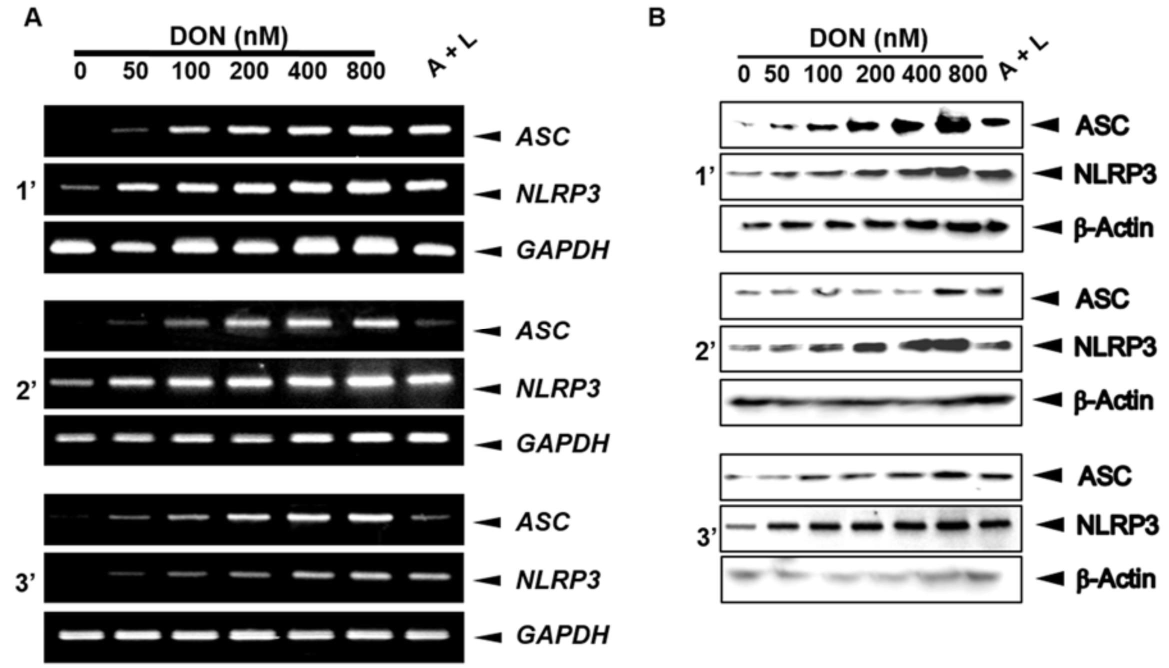


**Supplementary Figure 5:** These are all triplicate experiments for Figure 5. BV2 microglial cells were seeded at a density of 1 × $10^5$ cells/mL and treated with the indicated concentrations of DON or 1 mM ATP and 100 ng/mL LPS. (**A**) At 1 h after the administration of DON, the effect on *ASC* and *NLRP3* expression was assessed by RT-PCR. (**B**) The cytosolic fraction of BV2 microglial cell lysate was used to assess ASC and NLRP3 protein expression after 24 h.

EXCLI Journal 2019;18:356-369 – ISSN 1611-2156
Received: December 07, 2018, accepted: March 07, 2019, published: June 11, 2019

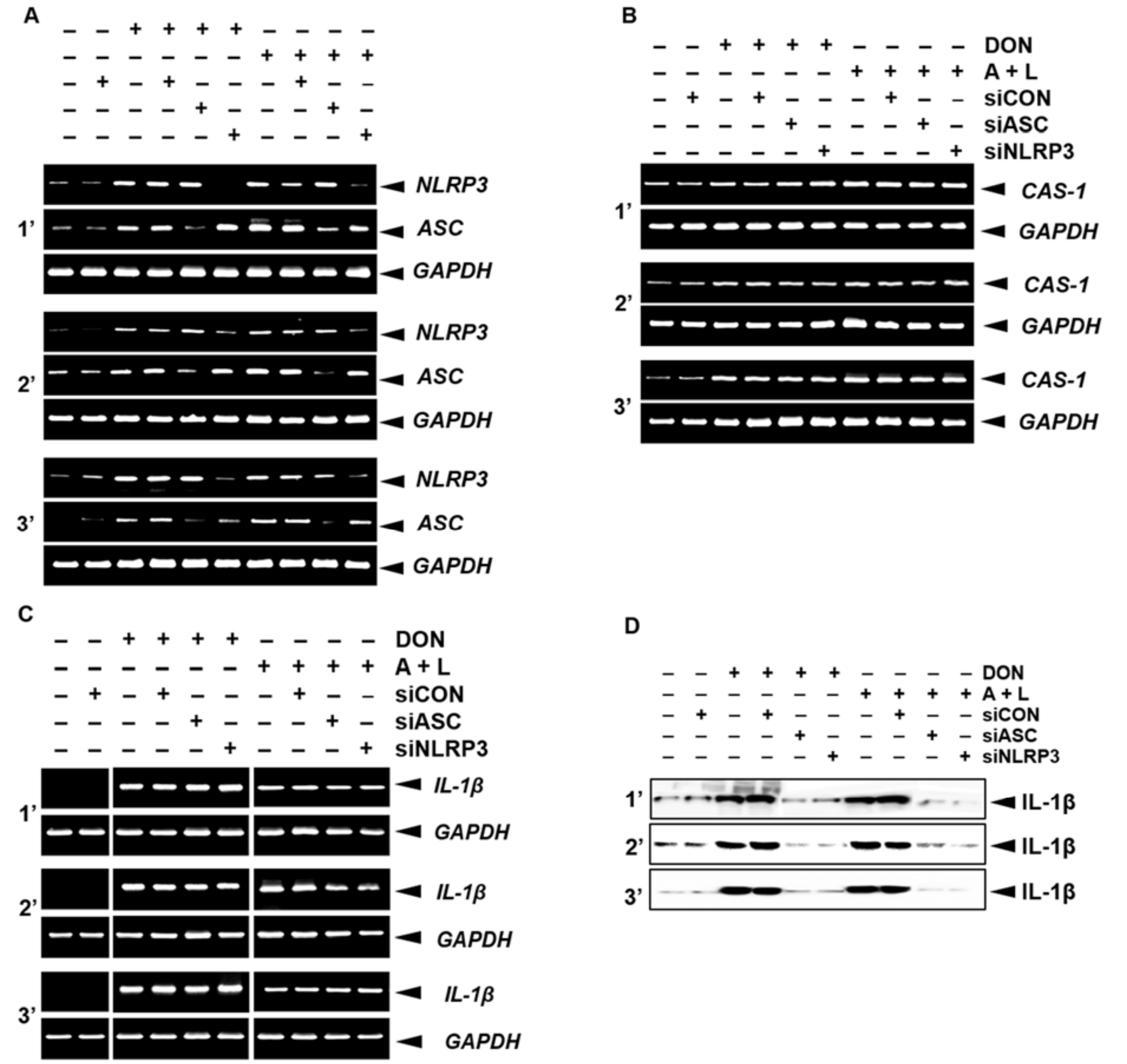


**Supplementary Figure 6:** These are all triplicate experiments for Figure 6. BV2 microglial cells were seeded at the density of 1 × $10^5$ cells/mL overnight and then transfected with siASC and siNLRP3 for 48 h. The cells were treated with DON (800 nM), or 1 mM ATP and 100 ng/mL LPS. (A) The effect of DON on ASC and NLRP3 expression was assessed by RT-PCR 1 h after treatment with DON, or ATP and LPS. (**B** and **C**) In a parallel experiment, caspase-1 (**B**) and pro-IL-1β (**C**) expression was detected by RT-PCR at samples taken 1 h after treatment. (**D**) The cytosolic expression of IL-1β was detected by western blotting at 24 h. CAS-1; caspase-1. A + L; treatment with 1 mM ATP and 100 ng/mL LPS.
